# Molecular Insight into Gene Response of Diorcinol- and Rubrolide-Treated Biofilms of the Emerging Pathogen Stenotrophomonas maltophilia

**DOI:** 10.1128/spectrum.02582-21

**Published:** 2022-04-26

**Authors:** Mirja Gudzuhn, Ifey Alio, Raphael Moll, Jessica de Vries, Jacob Boehlich, Maik Assmann, Jasmin Janneschütz, Nina Schützenmeister, Axel Himmelbach, Anja Poehlein, Rolf Daniel, Wolfgang R. Streit

**Affiliations:** a Department of Microbiology and Biotechnology, Universität Hamburg, Hamburg, Germany; b Department of Pharmaceutical Chemistry, University of Vienna, Vienna, Austria; c Department of Chemistry, Institute of Pharmacy, Universität Hamburg, Hamburg, Germany; d Leibniz Institute of Plant Genetics and Crop Plant Research (IPK), Seeland, Germany; e Institute of Microbiology and Genetics, Department of Genomic and Applied Microbiology, Georg-August University of Göttingen, Göttingen, Germany; Shenzhen Bay Laboratory

**Keywords:** antimicrobial activity, biofilms, transcriptome

## Abstract

Stenotrophomonas maltophilia is a multidrug-resistant human opportunistic pathogen. S. maltophilia contributes to disease progression in cystic fibrosis patients and is found in wounds and infected tissues and on catheter surfaces. Due to its well-known multidrug resistance, it is difficult to treat S. maltophilia infections. Strain-specific susceptibility to antimicrobials has also been reported in several studies. Recently, three fungal diorcinols and 14 rubrolides were shown to reduce S. maltophilia K279a biofilm formation. Based on these initial findings, we were interested to extend this approach by testing a larger number of diorcinols and rubrolides and to understand the molecular mechanisms behind the observed antibiofilm effects. Of 52 tested compounds, 30 were able to significantly reduce the biofilm thickness by up to 85% ± 15% and had strong effects on mature biofilms. All compounds with antibiofilm activity also significantly affected the biofilm architecture. Additional RNA-sequencing data of diorcinol- and rubrolide-treated biofilm cells of two clinical isolates (454 and K279) identified a small set of shared genes that were affected by these potent antibiofilm compounds. Among these, genes for iron transport, general metabolism, and membrane biosynthesis were most strongly and differentially regulated. A further hierarchical clustering and detailed structural inspection of the diorcinols and rubrolides implied that a prenyl group as side chain of one of the phenyl groups of the diorcinols and an increasing degree of bromination of chlorinated rubrolides were possibly the cause of the strong antibiofilm effects. This study gives a deep insight into the effects of rubrolides and diorcinols on biofilms formed by the important global pathogen S. maltophilia.

**IMPORTANCE** Combating Stenotrophomonas maltophilia biofilms in clinical and industrial settings has proven to be challenging. S. maltophilia is multidrug resistant, and occurrence of resistance to commonly used drugs as well as to antibiotic combinations, such as trimethoprim-sulfamethoxazole, is now frequently reported. It is therefore now necessary to look beyond conventional and already existing antimicrobial drugs when battling S. maltophilia biofilms. Our study contains comprehensive and detailed data sets for diorcinol and rubrolide-treated S. maltophilia biofilms. The study defines genes and pathways affected by treatment with these different compounds. These results, together with the identified structural elements that may be crucial for their antibiofilm activity, build a strong backbone for further research on diorcinols and rubrolides as novel and potent antibiofilm compounds.

## INTRODUCTION

Stenotrophomonas maltophilia is a Gram-negative multidrug-resistant bacterium. S. maltophilia is recognized as a clinically relevant human nosocomial opportunistic pathogen because it is associated mainly with respiratory infections, especially related to cystic fibrosis, but also with skin, blood, and catheter-related infections (1–6). Further it is found in environmental areas in association with roots and in fresh or wastewater (1). Notably, the species of S. maltophilia is extremely heterogeneous, consisting of at least 23 phylogenetic lineages (7, 8). This may, in part, explain the high phenotypic variety of its biofilm-forming ability and biofilm architecture on a population-wide level (9). Further it is noteworthy that S. maltophilia also shows high levels of phenotypic heterogeneity on a single-cell level (10).

Within this framework we demonstrated recently that the degree of virulence differs strongly between different clinical isolates independent of their phylogenetic position, biofilm-forming ability, or biofilm architecture (9).

S. maltophilia strains have been shown to be resistant to a wide range of antibiotics, and these resistant strains are heavily shielded with multiple efflux pumps and resistance genes such as β-lactamases (11, 12). Studies have reported that S. maltophilia biofilms display a decreased susceptibility to antibiotics (13, 14). As a result of this antibiotic resistance and the ability to form biofilms, infections caused by S. maltophilia in patients are extremely difficult to treat.

So far, many studies analyzed the antibiotic resistance of planktonic cells of S. maltophilia strains and isolates (15–22). Notably several studies already documented the occurrence of resistance against the commonly used antibiotic combination trimethoprim-sulfamethoxazole (TMP-SMX) to treat S. maltophilia infections (23–26). Further, some strains are resistant against the last-resort antibiotic colistin (polymyxin E) (27, 28).

Because of this increased multidrug resistance, alternative substances, like natural plant compounds (29–33), antimicrobial peptides (34), or the bacterial predator Bdellovibrio exovorus (35), have been studied to treat S. maltophilia infections. Our previous study revealed that some diorcinols inhibit the growth of Gram-positive bacteria, while they did not significantly inhibit the growth of Gram-negative bacteria (36). The same study also demonstrated for the first time that diorcinols can cause a 54% reduction of S. maltophilia K279a (here referred to as K279a) biofilms; therefore, diorcinols seem to be promising antibiofilm substances. Additionally, we also previously demonstrated that rubrolides had a significant antibiofilm activity against K279a (37).

**FIG 1:**
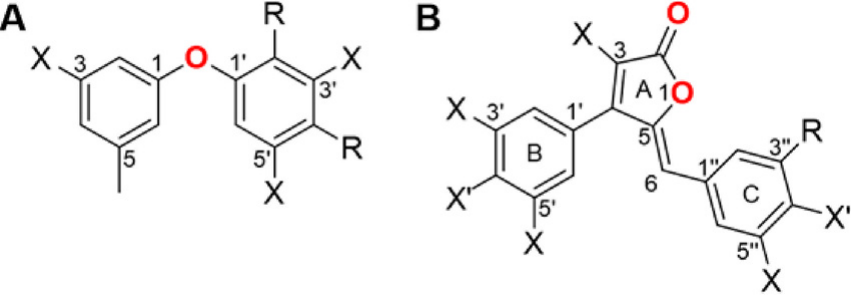
Basic structure of diorcinols and rubrolides used in this study. Carbon numbers and carbon rings are marked. (A) Basic diorcinol structure; X, methyl, hydroxyl, or methoxy group. R, possible site for 2-methyl-2-butene, 3-methyl-3-buten-2-one, or 3-methyl-3-buten-2-ol. (B) Basic rubrolide structure; X, possible halogenation; X′, hydroxyl group or halogen; R, possible sites for halogenation or 2,2-dimethyloxane (position 6-1), 2-methyl-2-butene, or 2-methyl-2-pentene.

Diorcinols are diphenyl ethers in which a hydroxy and methyl group (Fig. 1A) substitute both phenyl groups. They are mostly isolated from fungi (38–41) and are known to have antifungal (42) and antibacterial effects (36, 40, 43, 44). In addition, they have cytotoxic activity against tumorous cells (38, 39, 41). Similarly, rubrolides are metabolites most commonly isolated from various marine organisms (45–48) and fungi (49). Their basic structure consists of a central furanone ring flanked by two *para*-hydroxyphenyl moieties, while all rings can be halogenated (Fig. 1B). Several studies demonstrated an antibacterial activity against Gram-positive, but not against Gram-negative, bacteria (50, 51). Furthermore, rubrolides seem to have antiviral activity (37, 49, 52), and, similar to the diorcinols, some rubrolides have antitumor activity (46, 47, 49, 53). Interestingly, synthetic rubrolide analogues, but especially lactams derived from rubrolides, revealed antibiofilm activity against Gram-positive and Gram-negative bacteria (54–56).

Within these settings, we set out to test the effects of 52 synthetic diorcinols and rubrolides on a molecular level on two clinical isolates of S. maltophilia during biofilm formation. Thereby, we identified a few key genes linked to biofilm formation affected by these compounds. Furthermore, we define first structural elements linked to the design of diorcinols and rubrolides that seem to be required for their antibiofilm activity.

## RESULTS AND DISCUSSION

S. maltophilia is highly resistant against most antibiotics, and, as consequence, biofilms of clinical isolates are very difficult to treat. Recently, we showed that diorcinols and rubrolides strongly reduced biofilm formation of S. maltophilia K279a (36, 37). Intrigued by this initial and encouraging observation, we asked if we could identify individual genes and pathways involved in biofilm inhibition and what the impact was on the overall biofilm architecture and gene expression profiles of S. maltophilia biofilms. Further, we asked whether we could identify a link between diorcinol and rubrolide structural traits and the observed biofilm inhibitory effects.

For this purpose, we conducted laser scanning microscopy (LSM) imaging of biofilms exposed for 72 h to the various compounds. This analysis was conducted with 3 isolates, K279a, SKK 55, and 454. RNA-sequencing analysis of biofilm cells after treatment with diorcinols and rubrolides was also conducted with two isolates, K279a and 454.

### Diorcinols and rubrolides have strong antibiofilm activity against S. maltophilia.

**FIG 2:**
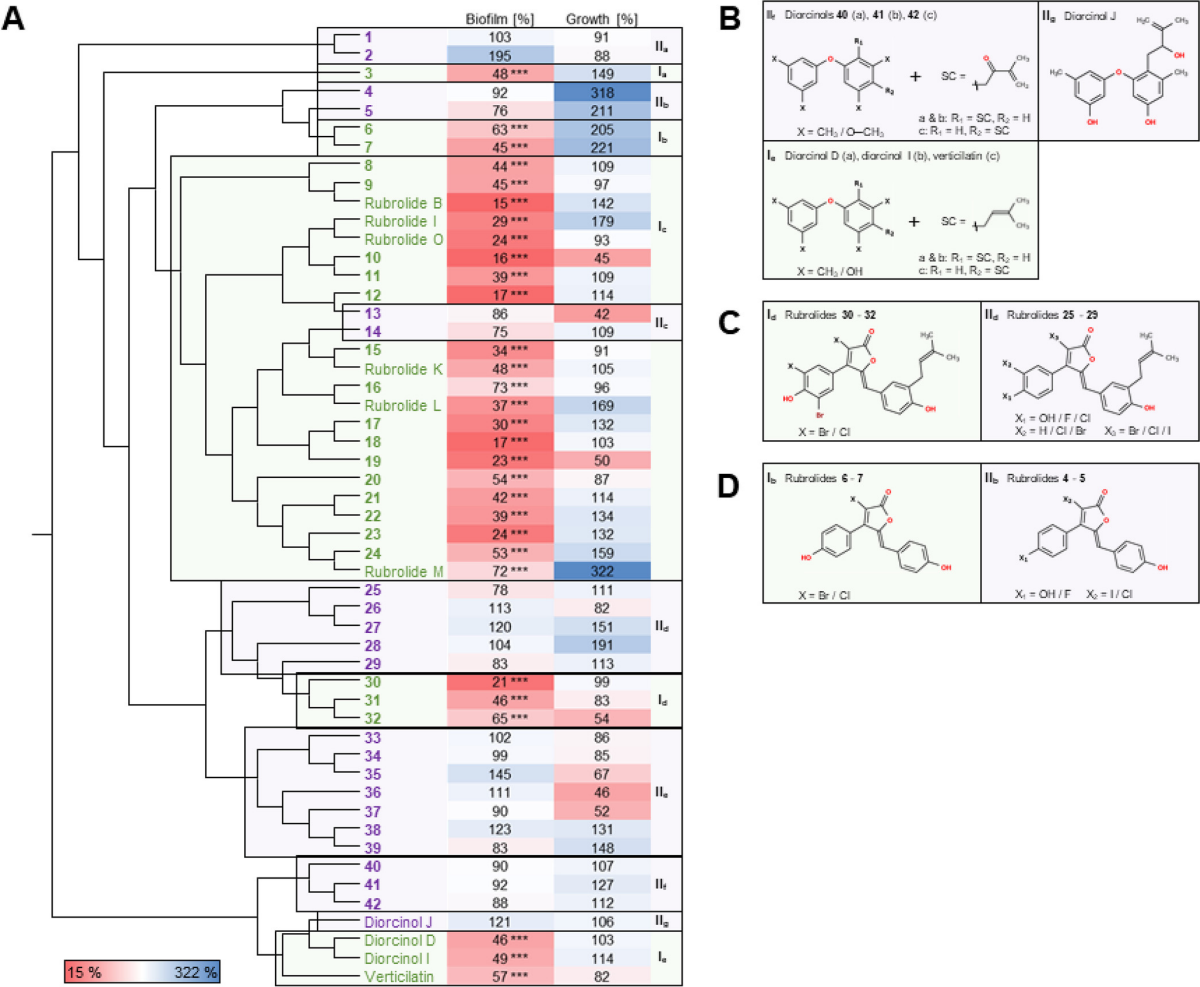
Clustering of synthetic diorcinols and rubrolides and their impact on biofilm formation and planktonic growth (OD) of K279a. (A) Hierarchical clustering of diorcinols and rubrolides calculated based on their structure was linked to the planktonic growth and biofilm formation of S. maltophilia K279a grown in the presence of 100 mg L^−1^ of the diorcinols and rubrolides analyzed in relation to the control (K279a grown with 2% DMSO). Cells were grown at 28°C in 10% LB medium for 24 h. The biofilm formation and planktonic growth of the control was set to 100%. Tests were done at least 3 times. Compounds in whose presence <75% of the control biofilm was formed were classified as antibiofilm effective (green; I_a–e_), while all other compounds were classified as noneffective (purple; II_a–g_). (B to D) Comparison of basic structure of selected antibiofilm effective versus noneffective compounds. All structures are illustrated in Fig. S1 in the supplemental material. Standard deviation of the biofilm formation ranged from 3.67 to 75.6% and for growth from 6.95 to 109.9%. Values can be found in Fig. S2; X, various elements; SC, side chain; ***, *P* < 0.001.

**FIG 3:**
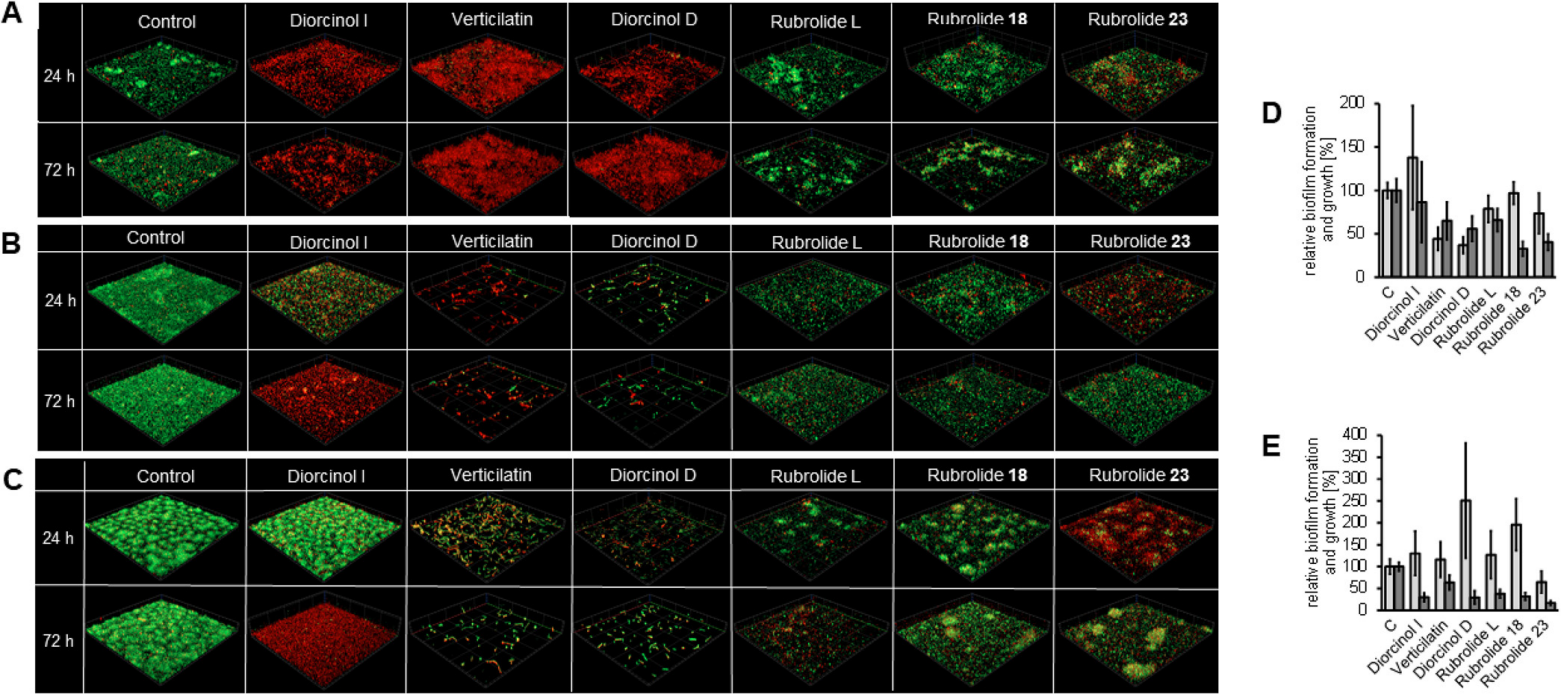
Diorcinols and rubrolides alter biofilm architecture of different S. maltophilia isolates. (A to C) The biofilm architecture of S. maltophilia K279a (A), SKK55 (B), and 454 (C) grown in the presence of 100 mg L^−1^ of the diorcinols and rubrolides was analyzed at different time points via CLSM after live/dead staining. Cells were grown in μ-slides at 28°C in 10% LB medium; red, dead cells; green, living cells. Images represent an area of 100 μm × 100 μm of the biofilm: control, biofilm grew in the presence of 1% DMSO. (D and E) Growth (light gray) and biofilm formation (dark gray) of S. maltophilia SKK55 (D) and 454 (E) grown in the presence of diorcinols and rubrolides were analyzed in relation to the control (cells grown in the presence of 1% DMSO) using crystal violet staining. Cells were grown at 28°C in 10% LB. Error bars indicate standard deviation. For SKK 55, *P* values ranged from <0.0001 to 0.4875 and from <0.0001 to 0.5786 for isolate 454.

To initially estimate the antibiofilm activity, we set out to analyze biofilm formation and planktonic growth of K279a grown in the presence of 7 diorcinols and 45 rubrolides (Fig. 2; Fig. S2 in the supplemental material). Verticilatin was not isolated from natural sources so far, since the structural analysis was incorrect and recently revised (36, 57). However, we decided to adhere to the given name “verticilatin” of the structure in this study to avoid confusion. All structures of the tested compounds are given in Fig. S1. Thirty substances had a strong impact on K279a biofilm formation based on crystal violet staining by revealing a ≥25% reduction of the film formation (Fig. 2A). The strongest effect on biofilm formation with 85% ± 15% reduction was achieved by rubrolide B, while the rubrolide analogue 16 revealed the lowest antibiofilm potential with a 27% ± 6% reduction. The diorcinols had slightly less pronounced impact on biofilm formation. The strongest reduction of biofilm thickness with 54% ± 12% was achieved by diorcinol D. An additional detailed analysis of the biofilm architectures implied that 21 rubrolides reduced and altered the biofilm architectures without killing the majority of the cells (Fig. S3). In contrast, diorcinols had a much stronger effect on K279a cell viability based on live/dead stains (Fig. 3; Fig. S3).

Based on these initial findings, we asked if other S. maltophilia isolates would also be affected by the rubrolide and diorcinol treatments. For this, we chose three diorcinols (diorcinol I, verticilatin, and diorcinol D) and three rubrolides (rubrolide L, 18, and 23). The effects of these compounds on S. maltophilia biofilms were tested for two additional clinical isolates, S. maltophilia SKK55 and 454, by analyzing biofilm architecture and cell viability (Fig. 3). As expected, the compounds altered the architecture and/or viability of biofilm cells when tested at concentrations of 100 mg L^−1^ (Fig. 3A to C). Notably, the planktonic growth of 454 was not reduced by any of the three tested diorcinols (Fig. 3E), while verticilatin and diorcinol D affected the growth of SKK55 by up to 63% ± 9% (Fig. 3D).

**FIG 4:**
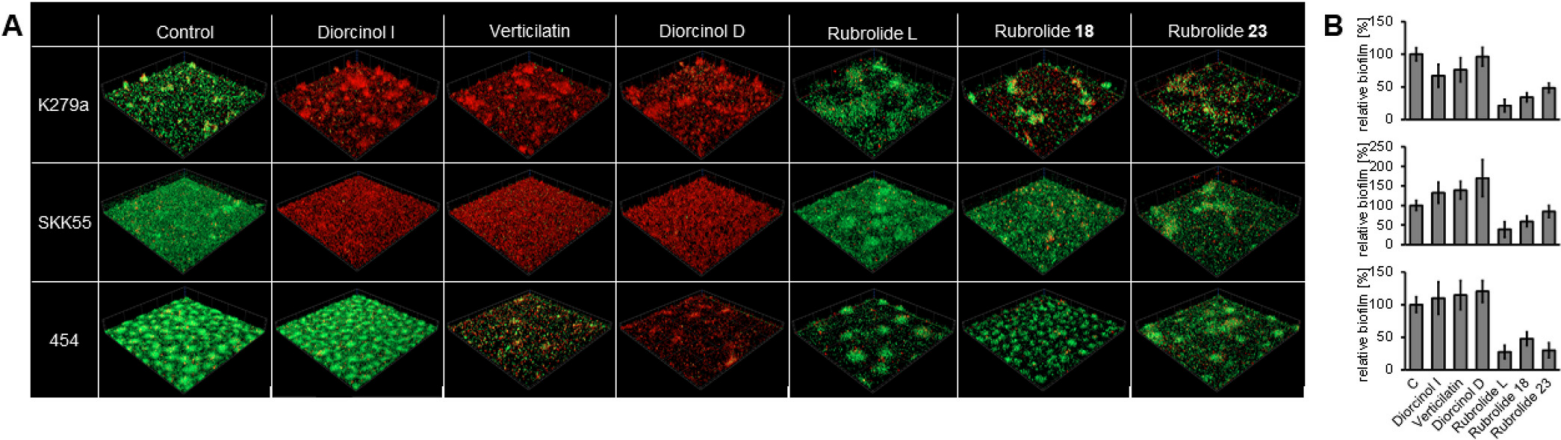
Diorcinols and rubrolides affect matured biofilms of different S. maltophilia isolates; 100 mg L^−1^ of diorcinols and rubrolides was added to a 24-h-old biofilm of S. maltophilia K279a, SKK55, and 454. (A) The biofilm architecture was analyzed with a CLSM after live/dead staining. Cells were grown in μ-slides at 28°C in 10% LB; red, dead cells; green, living cells. Images represent an area of 100 μm × 100 μm of the biofilm; control, biofilm grew in the presence of 1% DMSO. (B) The biofilm of S. maltophilia K279a, SKK55, and 454 grown in the presence of different diorcinols and rubrolides, which were added to a 24-h-old matured biofilm, was analyzed in relation to the control (cells grown in 1% DMSO) using crystal violet staining. Cells were grown at 28°C in 10% LB. Strain labeling of the graphs matches the labeling of A. Error bars indicate standard deviation. *P* values ranged from <0.0001 to 0.6119 for isolate K279a, from 0.0094 to 0.1242 for isolate SKK 55, and from 0.0004 to 0.5662 for isolate 454.

To further analyze whether any of the tested compounds would lead to detachment and dispersion of matured biofilms, they were added to 24-h-old biofilms of K279a, SKK55, and 454. Interestingly, matured biofilms of K279a and SKK55 appeared to contain a large fraction of dead cells after a 24-h treatment with the diorcinols (Fig. 4A). The biofilm architecture of K279a and SKK55 was not affected, indicating that diorcinols killed the cells but did not lead to a dispersion of the biofilm. This observation was confirmed by crystal violet staining of diorcinol-treated biofilms (Fig. 4B). Interestingly, diorcinol I did not have any effect on matured biofilms of 454, while verticilatin and diorcinol D strongly reduced biofilm thickness and altered its biofilm architecture (Fig. 4).

Since we used propidium iodine (PI) in our live/dead staining to visualize dead cells, the CFU/mL of K279a, SKK55, and 454 biofilms was determined to exclude cells that were accidentally stained with PI during microscopy due to permeabilized cell membranes. In these tests, the CFU/mL of the K279a, SKK55, and 454 biofilms grown in the presence of diorcinol I were 28% ± 17% to 96% ± 4% lower than the CFU/mL of the respective control biofilms (Fig. S4). For the K279a biofilm grown in the presence of verticilatin and diorcinol D, the CFU/mL was up to 93 ± 0.4% lower than the control, while no viable cells were detected for SKK55 and 454 biofilms. These data are in line with the visual inspections of the live/dead stains (Fig. 4).

Despite the studies that demonstrated an antibacterial effect for the diorcinols (36, 40, 43, 44), no study has so far analyzed their effect on biofilm architecture. Thus, the observations made here may imply that rubrolides and diorcinols are potentially interesting compounds for antibiofilm treatment.

### Structural elements of diorcinols and rubrolides responsible for antibiofilm activity.

Based on these observations, we asked if and to what extent different structural traits of the diorcinols and rubrolides were possibly linked to the strong antibiofilm response.

To identify the structural elements in the diorcinols and rubrolides leading to antibiofilm activity, a hierarchical clustering was calculated based on the structure of all substances (Fig. 2A). This clustering was linked to the relative (%) biofilm formation and planktonic growth of K279a in the presence of the different substances. Compounds in whose presence <75% of the control biofilm was formed were classified as antibiofilm effective (Fig. 2A, I_a–e_), while all other compounds were classified as noneffective (Fig. 2A, II_a–g_). As a result of this analysis, five structural clusters of compounds affecting biofilm formation were observed.

The analyzed diorcinols were placed into two structural clusters, which matched, except for diorcinol J, the classification of antibiofilm-effective and antibiofilm-noneffective substances. The antibiofilm-effective diorcinols were diorcinol D and I and verticilatin (Fig. 2A, I_e_). Diorcinol D and I had previously been shown to have antibacterial activity against the Gram-positive bacteria Staphylococcus aureus (44) and Enterococcus faecalis, while they were not effective against the Gram-negative bacteria Pseudomonas aeruginosa and Escherichia coli (36). Further analysis of the structural differences between effective and noneffective diorcinols identified a methyl or methoxy residue at position 3 and 3′ or 5′ of the noneffective diorcinols, while the effective diorcinols carry a methyl or hydroxyl group at the same positions (Fig. 2B). In contrast, the noneffective diorcinol J contains a hydroxyl group at these positions like the effective diorcinols but differs in the side chain, which is present in the structure of all diorcinols at position 2′ or 4′ in one of the phenyl groups. Diorcinol J has a hydroxyl group in the side chain (Fig. 2B, II_g_) in contrast to the effective diorcinols, while all other noneffective diorcinols have an oxygen at the same position in the side chain (Fig. 2B, II_f_). Notably, all the effective diorcinols possess a prenyl group as a side chain (Fig. 2B, I_e_), which may be responsible for the antibiofilm activity. This, however, needs to be verified with a larger number of molecule variants. Nevertheless, to our knowledge, just one study demonstrated an antibiofilm activity of diorcinol D and I and verticilatin against bacteria (36).

Few of the rubrolides, like rubrolides 25 to 32, carry the same side chain as diorcinol D, diorcinol I, and verticilatin at position 3′′ (Fig. 2C). However, this applies to antibiofilm-effective and antibiofilm-noneffective rubrolides and might imply that, at least for the rubrolides, the side chain is not the only relevant structural component leading to an antibiofilm activity. The effective rubrolides possessing this side chain have a bromine at position 5′ (Fig. 2C, I_d_), which is missing in the structure of noneffective rubrolides (Fig. 2C, II_d_). Generally, a bromine or chlorine in the rubrolide structure could be a good indicator for an effective rubrolide, since the effective rubrolides 6 and 7 possess either a bromine or chlorine at position 3 of the furanone ring (Fig. 2D, I_b_); 71% of all tested rubrolides have this basic structure. However, the structurally most similar compounds are not effective against the biofilm of K279a (rubrolide 4 and 5, Fig. 2D, II_b_). Manzanaro et al. demonstrated that a chlorination of the central furanone significantly increases the inhibitory activity of rubrolides toward the human aldose reductase (ADL2) (58). Furthermore, they detected a correlation between the inhibitory activity and the bromination degree of nonchlorinated rubrolides, while this was not observed for chlorinated rubrolides. Contrary to their data, we observed an increasing antibiofilm activity against K279a in correlation with an increasing bromination degree of rubrolides possessing a chlorine in the furanone ring (Table 1). However, there was no general correlation between the overall total amount of bromines of nonchlorinated rubrolide and the antibiofilm activity. This indicates that the halogenation grade itself is not decisive for antibiofilm activity but a certain combination and/or position of halogens within the structure.

**TABLE 1:** Correlation between bromination degree of chlorinated rubrolides and proportional biofilm formation

Rubrolide ID	∑ Br in B ring^*a*^	∑ Cl in A ring^*a*^	∑ Br in C ring^*a*^	∑ Br total^*a*^	Biofilm formation (%)^*b*^
MA330	0	1	0	0	45
Rubrolide M	0	1	1	1	72
MA126	1	1	0	1	53
Rubrolide K	1	1	1	2	48
Rubrolide L	0	1	2	2	37
MA127	2	1	0	2	30
Rubrolide I	1	1	2	3	29
Rubrolide O	2	1	1	3	24
Rubrolide B	2	1	2	4	15

a ∑, sum of atoms; Br, bromine; Cl, chlorine.

b Percentage of biofilm formation of S. maltophilia K279a grown in the presence of different rubrolides in relation to the control (K279a grown with 1% DMSO).

In contrast to the high proportion of compounds (57.7%) affecting the biofilm of K279a, just 13.5% (7 substances) of the compounds, which belong all to the rubrolides, reduced the growth of K279a planktonic cultures (Fig. 2A; Fig. S2). This leads to the assumption that most of the substances affect the biofilm but do not affect the planktonic growth of K279a.

### Global transcriptome analysis identifies a set of differentially regulated genes in S. maltophilia biofilms grown in the presence of diorcinols and rubrolides.

In addition to the above observations with respect to the biofilm inhibitory effects, we asked to what extent the most effective compounds would have an influence on the level of gene expression in S. maltophilia biofilms. To get a first impression on the gene expression of biofilms exposed to these effective compounds, we decided to use only 2 isolates. For this, biofilms formed by the isolates K279a and 454 were chosen; isolate 454 was recently described as a rather virulent strain (9), and K279a was chosen as it is the model strain. Bearing in mind that having only 2 isolates is a limitation for this study, analysis of strongly regulated genes will be followed up by quantitative real-time PCR (qPCR) in other isolates. The overall gene expression profiles were analyzed for the three diorcinols (diorcinol I, diorcinol D, and verticilatin) and the three rubrolides (rubrolide L, 18, and 23); 1% dimethyl sulfoxide (DMSO) was used as a negative control.

While we observed that the rubrolides did not strongly affect cell viability, the diorcinols had a severe impact (Fig. 3A). Nevertheless, we extracted RNA from mature biofilms and used it for the RNA-sequencing analyses independent from the viability status of the cells.

### Transcriptomics analyses imply a small set of coregulated genes in treated biofilms.

Within these settings, the RNA-sequencing data implied that, in general, 16.3% of the genes of K279a and 28.6% of the genes of 454 were differently regulated after treatment with diorcinols, whereas 2.81% of the genes of K279a and 1.81% of the genes of 454 were differently regulated after treatment with rubrolides using a log_2_ fold change cutoff of 2 and −2 and an adjusted *P* value of ≤0.05 (Tables 2 and 3).

**TABLE 2:** Shared upregulated genes in K279a and 454 after diorcinol treatment of biofilm cells

Locus tag	Protein ID	Annotation	Log_2_ fold change	*P* value
SMLT_RS22205	WP_005414956.1	STAS domain-containing protein	2.21–2.87	1.98E−11 to 1.27E−19
SMLT_RS19365	WP_005411088.1	Multidrug efflux RND transporter permease subunit SmeE	3.42–7.20	3.09E−15 to 1.53E−27
SMLT_RS02565	WP_005407892.1	Hypothetical protein	2.80–5.12	3.57E−07 to 3.71E−19
SMLT_RS19360	WP_012481298.1	Multidrug efflux RND transporter outer membrane subunit SmeF	3.98–8.31	8.58E−14 to 2.84E−22
SMLT_RS03005	WP_012479067.1	NAD-dependent epimerase/dehydratase family protein	2.31–5.33	1.72E−12 to 4.28E−32
SMLT_RS04345	WP_004145339.1	50S ribosomal protein L2	2.00–3.52	7.56E−10 to 3.31E−26
SMLT_RS16165	WP_005410456.1	NADH-quinone oxidoreductase subunit NuoI	2.18–5.12	1.63E−15 to 1.70E−30
SMLT_RS04505	WP_004153634.1	Malate dehydrogenase	2.93–4.07	4.45E−18 to 2.35E−51
SMLT_RS05065	WP_012479298.1	Phage tail sheath subtilisin-like domain-containing protein	2.90–5.47	1.15E−07 to 2.12E−26
SMLT_RS16160	WP_010486441.1	NADH-quinone oxidoreductase subunit J	2.40–6.23	3.17E−21 to 1.60E−44
SMLT_RS08685	WP_005409079.1	Succinate dehydrogenase iron-sulfur subunit	2.44–4.49	8.39E−19 to 4.14E−33
SMLT_RS15225	WP_005410273.1	Dihydrolipoyl dehydrogenase	2.54–3.47	8.25E−15 to 5.82E−35
SMLT_RS05090	WP_005408332.1	GPW/gp25 family protein	2.29–5.68	0.000153601 to 3.65E−05
SMLT_RS02570	WP_012479008.1	Hypothetical protein	3.19–6.01	2.21E−08 to 5.75E−22
SMLT_RS16155	WP_005410454.1	NADH-quinone oxidoreductase subunit NuoK	2.33–6.42	7.75E−12 to 6.69E−22
SMLT_RS17870	WP_005410793.1	Succinate-CoA ligase subunit alpha	2.12–3.08	1.53E−15 to 2.72E−31
SMLT_RS22210	WP_012481677.1	VacJ family lipoprotein	2.12–4.31	2.21E−28 to 7.72E−43
SMLT_RS22120	WP_012481665.1	ATP-binding protein	2.51–6.66	6.64E−08 to 2.32E−47
SMLT_RS12545	WP_005409784.1	Hypothetical protein	2.16–4.56	5.44E−05 to 2.48E−20

**TABLE 3:** Shared downregulated genes in K279a and 454 after diorcinol treatment of biofilm cells

Locus tag	Protein ID	Annotation	Log_2_ fold change	*P* value
SMLT_RS01840	WP_005407753.1	LysR family transcriptional regulator	−2.20 to −3.81	4.39E−10 to 5.15E−25
SMLT_RS02610	WP_005407901.1	TetR/AcrR family transcriptional regulator	−2.76 to −4.65	1.50E−15 to 3.29E−37
SMLT_RS10145	WP_012479990.1	DNA-binding protein	−2.23 to −4.44	7.51E−09 to 9.35E−23
SMLT_RS15115	WP_005410251.1	Metalloregulator ArsR/SmtB family transcription factor	−2.11 to −5.19	2.49E−09 to 7.35E−56
SMLT_RS10390	WP_012480030.1	ATP phosphoribosyltransferase	−2.88 to −7.52	1.31E−06 to 4.77E−31
SMLT_RS10385	WP_005416326.1	Helix-turn-helix domain-containing protein	−3.09 to −9.61	1.70E−11 to 1.49E−54

**FIG 5:**
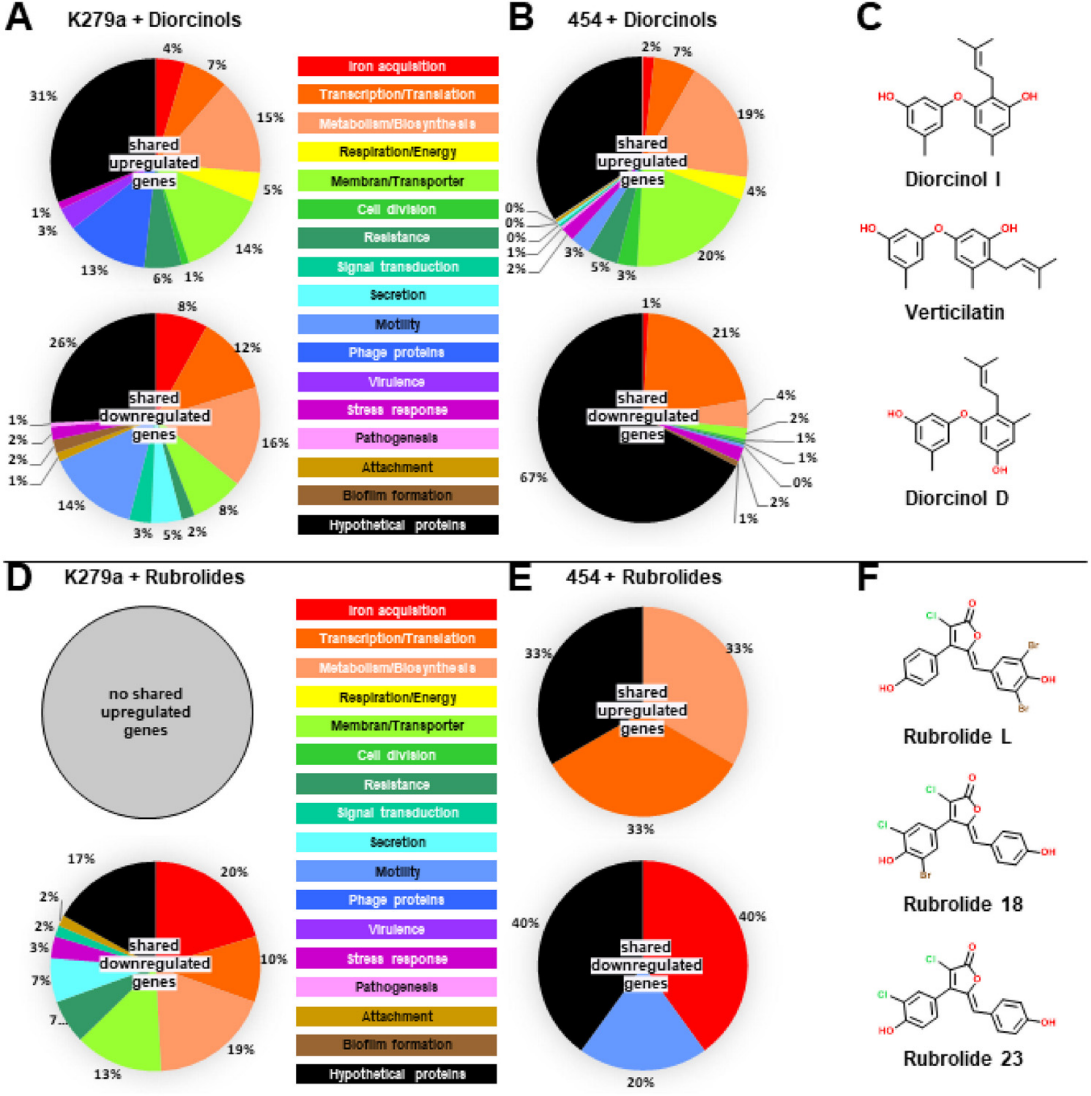
Shared regulated genes in K279a and 454 after treatment with diorcinols and rubrolides for 72 h. (A to C) Shared up- and downregulated genes of K279a (A) and 454 (B) after treatment with diorcinol I, diorcinol D, and verticilatin (C) for 72 h. (D to F) Shared up- and downregulated genes of K279a (D) and 454 (E) after treatment with rubrolide L, 18, and 23 (F) for 72 h.

In general, the most strongly regulated genes in both treatments and in both strains were linked to iron acquisition, drug extrusion, metabolic pathways, membrane proteins, and secretion processes (mainly secretion system 2 [T2SS]). The largest pool of differentially regulated genes had no predicted function and were assigned as hypothetical proteins (Fig. 5; Tables S2 and S3).

A more detailed analysis indicated that especially the diorcinols had a strong impact on the transcription of phage assembly genes and cell membrane biosynthesis (Table S2). Prophages have been shown to contribute to drug resistance in bacteria (59), and their induction is generally correlated with stress response (60, 61).

Notably, 19 genes were commonly upregulated by diorcinol treatment in both strains across all three different molecules (Fig. 5A and B; Tables S2 and S4). In general, upregulated genes in the diorcinol treatment were affiliated with cell wall and lipopolysaccharide (LPS) biosynthesis, drug extrusion, and iron acquisition (Table S2). The upregulation of several drug efflux pumps and resistance mechanisms implies that the diorcinols might be able to enter the cells, leading to activation of resistance mechanisms and transporters.

### Motility, metabolism, and transport are the main targets of diorcinol-activated gene expression.

Among the most strongly downregulated genes in K279a treated with diorcinols, some genes were found that are involved in motility and flagella biosynthesis, metabolism, and membrane transport (Fig. 5A; Table S2). Notably, the outer membrane protein A (OmpA), commonly involved in bacterial drug resistance, was strongly (log_2_ fold change of −10.73) downregulated in 454 biofilms treated with diorcinols (Table S2). OmpA has a broad range of functions in different microorganisms. It is a key virulence factor, which mediates bacterial biofilm formation, eukaryotic cell infection, antibiotic resistance, and immunomodulation (62). OmpA has been reported to function as an adhesin and invasin and participates in biofilm formation in Escherichia coli, Sodalis glossinidius, and Acinetobacter baumannii (63–68). It also serves as a receptor that binds to some bacteriophages (69). Only few functions of OmpA are known in S. maltophilia. A study by Liao and colleagues demonstrated that the interplay between OmpA and RpoN regulates flagellar synthesis in S. maltophilia (70).

Since a dysregulation and permeabilization of the membrane by diorcinol D was reported for Candida albicans (71), the regulation of membrane proteins in S. maltophilia biofilms treated with diorcinols might indicate that they have a similar effect on its membrane.

### AX21 homologues and cyclic di-GMP (c-di-GMP) appear to play a role in diorcinol-treated cells.

Interestingly, an AX21 family protein was upregulated in K279a biofilms treated with all three diorcinols (log_2_ fold change of 3 to 3.9; Table S2). AX21 family proteins are located in the membrane of outer membrane vesicles and are probably related to biofilm formation and virulence in S. maltophilia (72). Further, genes related to biofilm formation were found to be regulated during the treatment with diorcinols or rubrolides. One of these genes encodes a c-di-GMP phosphodiesterase and was upregulated in all diorcinol treatments of 454 biofilms (log_2_ fold change of 2 to 5; Table S2). The c-di-GMP phosphodiesterase catalyzes the hydrolysis of c-di-GMP. Since a low intracellular level of c-di-GMP is a signal for biofilm dispersion (73), the observation made here may explain the relatively strong antibiofilm effects of diorcinols. A further factor leading to this effect might be the downregulation of the biofilm growth-associated repressor STMAn7_19620 in 454 biofilms treated with all diorcinols (Table S2).

This finding also might give first hints that diorcinols directly or indirectly affect the transcription machinery, especially because several further transcription regulators were regulated in K279a and 454 in response to a treatment with diorcinols.

### Rubrolides mainly affect TonB-like transport and T2SS.

Interestingly, no shared regulated genes across both strains and all treatments were found after the rubrolide treatment. Among the top 10 downregulated genes in biofilms of both strains exposed to rubrolides, some genes were affiliated with *tonB*-like transporters, which are linked to iron, magnesium, and in general cation uptake, T2SS, metabolic pathways, and membrane proteins (Fig. 5D and E; Tables S3 and S4). In addition, hemolysin secretion was significantly downregulated as was a YgsS family enzyme, which is involved in pyridoxal 5-phosphate homeostasis.

The obtained transcriptomic data gave a deep insight into the metabolism of S. maltophilia biofilms treated with rubrolides and diorcinols. Clearly, our transcriptome data did not immediately deliver single and specific target genes for each of the tested strains and the tested substances. However, the RNA-sequencing data imply that the rubrolides and diorcinols have a strong impact on general metabolic pathways, energy maintenance (i.e., cyclo di-GMP levels), cell wall and membrane biosynthesis, and transport processes (i.e., ompD; Fig. 5). These metabolic routes appear to be the primary targets of diorcinols and rubrolides on S. maltophilia biofilms. Future work will have to carefully evaluate if these substances can be used to treat S. maltophilia biofilms in clinical or industrial settings. Within this framework, it is noteworthy that no study has shown a cytotoxicity of diorcinols and rubrolides toward healthy human cell lines. Thereby, Pearce et al. demonstrated that rubrolide O had no significant short-term toxicity to human neutrophils at a concentration of 500 μM by showing only 6% lower cell viability than the control (47). Since a concentration of 100 mg L^−1^ of rubrolide O, corresponding to a concentration of 181.4 μM, reduced the biofilm formation of K279a by 76% ± 16% (Fig. 2A; Fig. S2), which is well below the concentration tested by Pearce et al., toxicity to human cells might be unlikely. Furthermore, rubrolide E, F, R, and S were shown to have anti-inflammatory activity, while they did not show cytotoxic activity at the same concentration of 10 μM (74). However, a cytotoxic activity of rubrolides against cancer cells was detected (46, 47, 49, 53), but whether the effect on cancer cells differs from the effect on healthy cells remains unknown so far and has to be analyzed in the future.

### Conclusions.

This study presents a detailed analysis of the effects of rubrolides and diorcinols on S. maltophilia biofilm cells. A hierarchical clustering of all 52 used compounds and their structures revealed that the strong antibiofilm activity of rubrolide B is probably due to the chlorination of the furanone and a high degree of bromination. This, however, needs to be confirmed with a higher number of molecules. Furthermore, this clustering discovered that the presence of a prenyl group in the side chain of diorcinols results in antibiofilm-active compounds, which even kills cells in the deeper biofilm layers. By contrast, treatment with rubrolides led to a more porous biofilm, which might indicate that rubrolides specifically affect and reduce biofilms without substantially killing the cells. The comprehensive RNA-sequencing data set of diorcinol- or rubrolide-treated biofilms gives a deep insight into the physiology of the bacteria treated with these compounds. While no direct novel drug target was identified, the data give first clues on potential targets linked to cell wall biosynthesis, energy metabolism, transport, and secretion processes. Altogether this study will lay the foundation for further research on diorcinols and rubrolides as potential antibiofilm compounds in Gram-negative bacteria.

## MATERIALS AND METHODS

### Bacterial strains, chemicals, and growth conditions.

Table S1 in the supplemental material summarizes the bacterial strains and isolates used in this study. All strains were routinely cultured in LB medium (10 g/L tryptone, 5 g/L yeast extract, and 5 g/L NaCl) at 28°C or 37°C if not otherwise stated. Diorcinols and rubrolides were used in concentrations ranging from 0 to 100 mg L^−1^ and were provided by the Department of Chemistry, Institute of Pharmacy, Universität Hamburg. Colistin was dissolved in H_2_O, and the tested substances were dissolved in 100% DMSO.

### Biofilm assays.

For antibacterial and antibiofilm testing of the compounds, the overnight culture of S. maltophilia was adjusted to 4.0 × 10^7^ cells/mL. Two hundred microliters of the diluted culture was pipetted in microtiter plates (Nunc MicroWell, flat and U bottom, Thermo Fisher Scientific, Waltham, MA, USA) supplemented via a dilution series with the appropriate concentration of the substance up to 100 mg L^−1^. The negative control contained 1 to 2% of the solvent (DMSO). Biofilm formation was analyzed via crystal violet staining after 24 h of growth as previously described (9).

To analyze the antibiofilm activity of compounds on a matured biofilm, biofilms were grown for 24 h before the supernatant was removed, and fresh medium supplemented with the compounds was carefully added. Biofilm reduction was analyzed via crystal violet staining after a further 24 h of growth. All assays were performed in triplicate with each six biological replicates.

### Fluorescence imaging analysis of biofilms.

Biofilms were cultivated as previously described (9). Overnight cultures of S. maltophilia were adjusted to 4.0 × 10^7^ cells/mL. The medium was supplemented with the appropriate substance and the negative control with 1 to 2% of the solvent (DMSO). Cells were grown in μ-slide 8-well plates (ibiTreat, 80826, ibidi USA Inc., Fitchburg, Wisconsin). Visualization of μ-slide biofilms was performed using a confocal laser scanning microscope (CLSM) as previously described (9). Therefore, cells were stained with the LIVE/DEAD BacLight bacterial viability kit (Thermo Fisher Scientific, Waltham, MA, USA).

### Growth analysis.

For the growth analysis, an overnight culture was adjusted to 4.0 × 10^7^ cells/mL in 50 mL of 10% LB medium supplemented with 100 mg L^−1^ of the appropriate substance; 1% DMSO was used as a negative control. Growth was monitored for up to 24 h by measuring the optical density at 600 nm (OD_600_) every 30 min. For each treatment, three biological replicates were done.

### CFU/mL determination.

An overnight culture was adjusted to 4.0 × 10^7^ cells/mL in 10% LB medium supplemented with 100 mg L^−1^ of the appropriate substance; 1% DMSO was used as a negative control. Six milliliters per well of the diluted culture supplemented with the appropriate substance was pipetted into 6-well plates (Nunc MicroWell, 142475, Thermo Fisher Scientific, Waltham, MA). The biofilms grew under static conditions at 28°C for 24 h. Afterward, the supernatant was discarded, and the biofilm was resuspended in 3 mL of LB medium per tested condition. The cells were pelleted at 4°C for 15 min, and the pellets were resuspended in 3 mL of LB medium, respectively. The cell suspensions were adjusted to 4.0 × 10^7^ cells/mL in LB medium, and a dilution series up to 10^−6^ was prepared. Dilution steps 10^−4^ to 10^−6^ were plated on LB plates. Colonies were counted, and CFU/mL was determined after overnight incubation at 37°C. For each treatment, a minimum of biological replicates were analyzed.

### RNA sequencing and data analysis.

Overnight cultures of S. maltophilia clinical isolates (K279a and 454) were adjusted to 4.0 × 10^7^ cells/mL. The biofilms grew in 24-well microtiter plates (Nunc MicroWell, 142475, Thermo Fisher Scientific, Waltham, MA) in 10% LB at 28°C for 72 h in the presence of 100 mg L^−1^ of the appropriate substance; 1% DMSO was used as a negative control. For the preparation of cell material for RNA sequencing, biofilms were harvested with a 20% stop mix (stop mix: 95% ethanol and 5% phenol) and washed with phosphate-buffered saline (PBS). Pellets were frozen in liquid nitrogen until analysis. The transcriptomes of a total of 12 different biofilm samples were analyzed. Since for each analysis three independent biological replicates were used, a total of 36 samples were finally processed. Triplicate samples treated with 100 mg L^−1^ of six different compounds were analyzed.

Harvested biofilms were resuspended in 800 μL of RLT buffer from the RNeasy minikit (Qiagen, Hilden, Germany) with β-mercaptoethanol (10 μL/mL), and cell lysis was achieved using a laboratory ball mill. Afterwards, 400 μL of RLT buffer (RNeasy minikit) with β-mercaptoethanol (10 μL/mL) and 1,200 μL of 96% (vol/vol) ethanol was added. The RNeasy minikit was used as recommended by the manufacturer for RNA isolation, but instead of RW1 buffer, RWT buffer (Qiagen, Hilden, Germany) was added to also isolate RNAs smaller than 200 nt. To determine the RNA integrity number (RIN), the isolated RNA was run on an Agilent Bioanalyzer 2100 with an Agilent RNA 6000 Nano kit as recommended by the manufacturer (Agilent Technologies, Waldbronn, Germany). Remaining genomic DNA was eliminated by treating the samples with TURBO DNase (Thermo Fisher Scientific, Waltham, MA, USA). The Illumina Ribo-Zero plus rRNA depletion kit (Illumina Inc., San Diego, CA, USA) was used to reduce the amount of rRNA-derived sequences. For sequencing, the strand-specific cDNA libraries were created with an NEBNext Ultra II directional RNA library preparation kit for Illumina (New England BioLabs, Frankfurt am Main, Germany) using 50 ng of rRNA-depleted RNA and 8 PCR cycles. To determine the quality and size of the libraries, samples were run on an Agilent Bioanalyzer 2100 using an Agilent high sensitivity DNA kit as recommended by the manufacturer (Agilent Technologies, Waldbronn, Germany). Concentrations of the libraries were analyzed using the Qubit dsDNA HS assay kit following the manufacturer’s instructions (Life Technologies GmbH, Darmstadt, Germany). Sequencing was performed on the NovaSeq 6000 instrument (Illumina Inc., San Diego, CA, USA) using NovaSeq 6000 SP reagent kit v1.5 (100 cycles; paired-end 2 × 61 cycles and 2 index reads with 8 cycles each) and the NovaSeq XP 2-Lane kit v1.5.

For quality filtering and discarding of remaining adaptor sequences, Trimmomatic-0.39 (75) and a cutoff phred-33 score of 15 were used. Mapping against the reference genomes was performed with Salmon (v 1.5.2) (76). As a mapping backbone, a file that contained all annotated transcripts excluding rRNA genes and the whole genome of the references as decoy was prepared with a k-mer size of 11. Decoy-aware mapping was done in selective-alignment mode with “–mimicBT2,” “–disableChainingHeuristic,” and “–recoverOrphans” flags as well as sequence and position bias correction. For –fldMean and –fldSD, values of 325 and 25 were used, respectively. The quant.sf files produced by Salmon were subsequently loaded into R (v 4.0.3) (77) using the tximport package (v 1.18.0) (78). DeSeq2 (v 1.30.0) (79) was used for normalization of the reads, and foldchange-shrinkages were also calculated with DeSeq2 and the apeglm package (v 1.12.0) (80). Genes with a log_2_ fold change of +2/−2 and an adjusted *P* value of <0.05 were considered differentially expressed. Per condition, three biological triplicates have been sequenced and analyzed.

### Hierarchical clustering.

Hierarchical clustering based on the structures of the diorcinols and rubrolides was performed with ChemMine tools (https://chemminetools.ucr.edu/). The distance matrix was generated by subtracting the similarity measure (Tanimoto coefficient) from one. This matrix was the input for the clustering by using the hclust function, which hierarchically joins the most to least similar items by using the single linkage method (81).

### Data availability.

For the 27 analyzed biofilm samples, we retrieved between 10.4 and 29.1 million raw reads, ensuring a sufficiently high coverage. The trimmed reads have been deposited in the National Center for Biotechnology Information’s (NCBI) Sequence Read Archive (SRA) under the BioProject ID PRJNA783225 and SRA accession numbers SRR17028240 to SRR17028266.
