# Supplementary material for: Molecular Insight into Gene Response of Diorcinol- and Rubrolide-Treated Biofilms of the Emerging Pathogen Stenotrophomonas maltophilia
Source: Microbiol Spectr. 2022 Apr 26;10(3):e02582-21. doi: 10.1128/spectrum.02582-21 (PMC9241881; doi:10.1128/spectrum.02582-21)
Supplement: SUPPLEMENTAL FILE 1 — Supplemental material. Download spectrum.02582-21-s001.pdf, PDF file, 1.1 MB [file spectrum.02582-21-s001.pdf]

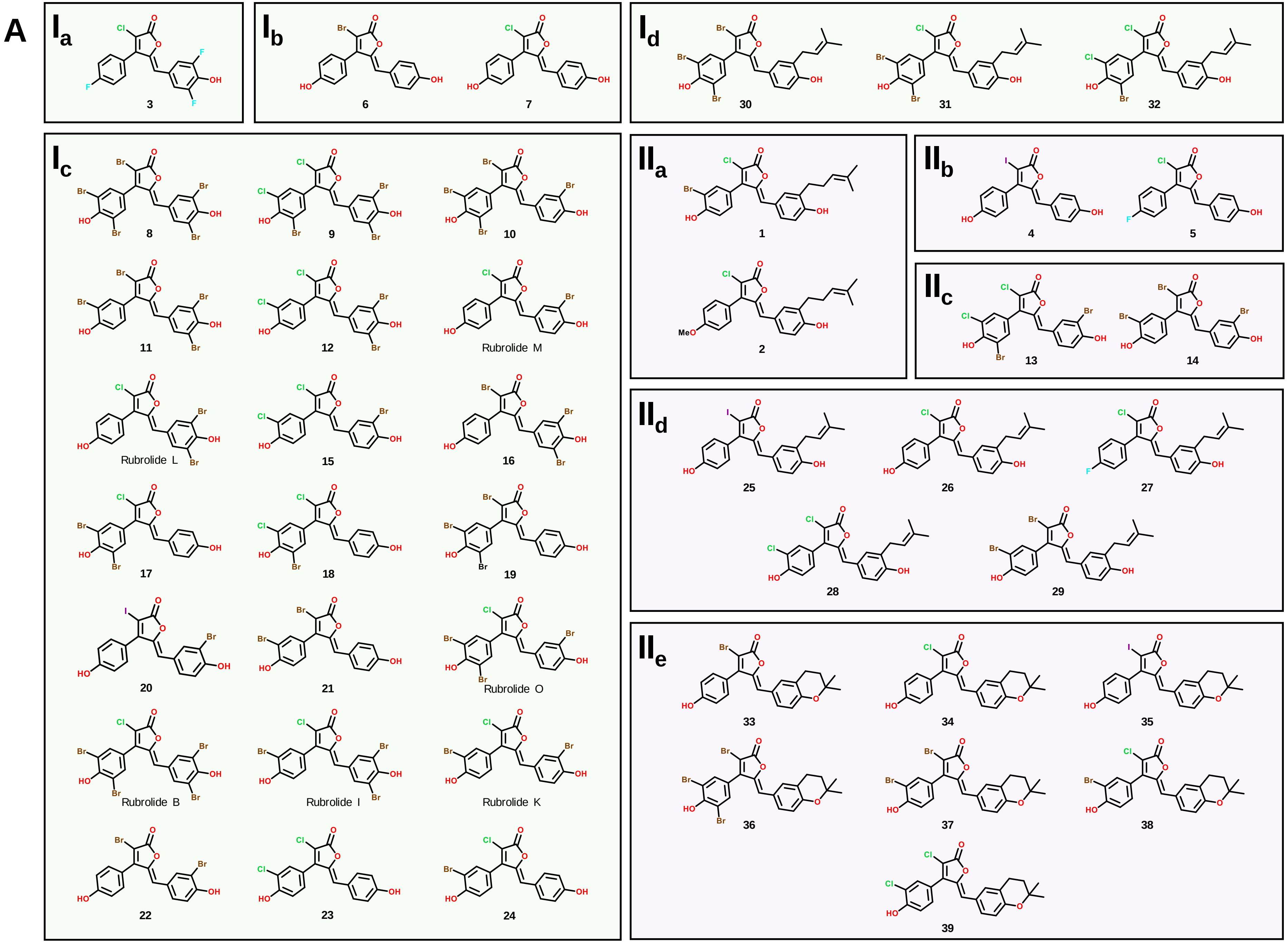

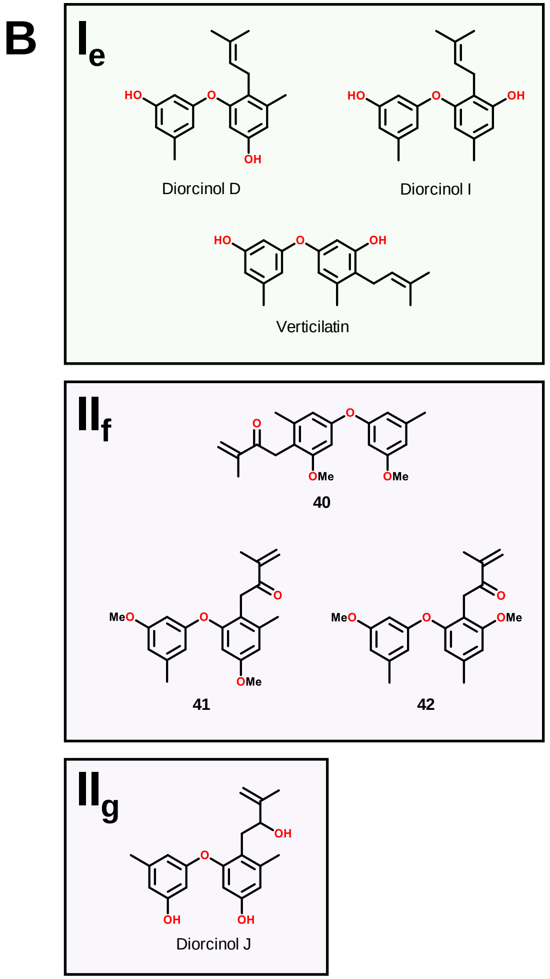

Figure S1: Structures of all diorcinols and rubrolides used in this study. Structures are grouped into antibiofilm effective (Green; $I_{a\text{-}e}$) and non-effective (Purple; $II_{a\text{-}g}$) according to hierarchical clustering (Figure 2A).

FIGURE S1

FIGURE S2

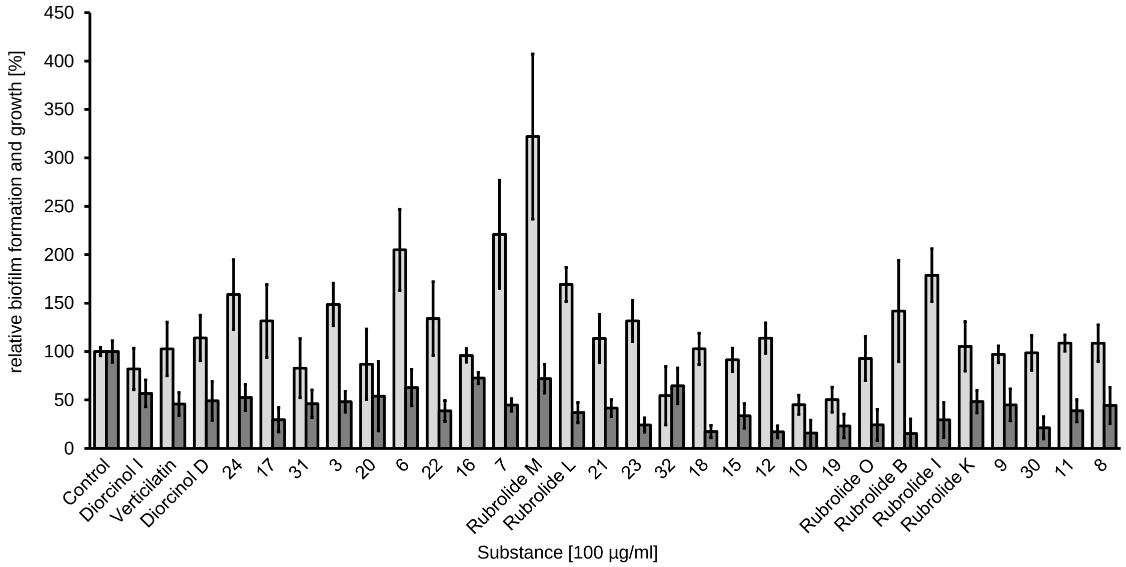

FIGURE S2: Diorcinols and rubrolides reduce the biofilm formation of *S. maltophilia* K279a. The planktonic growth (light grey) and biofilm formation (dark grey) of *S. maltophilia* K279a grown in presence of 100 mg L^{-1} of the diorcinols and rubrolides were analyzed in relation to the control via crystal violet staining. Planktonic cells were grown at 28 °C in 10 % LB medium. C = control (K279a grown with 2 % DMSO). Error bars indicate standard deviation. Data represent mean values of at least 3 replicates.

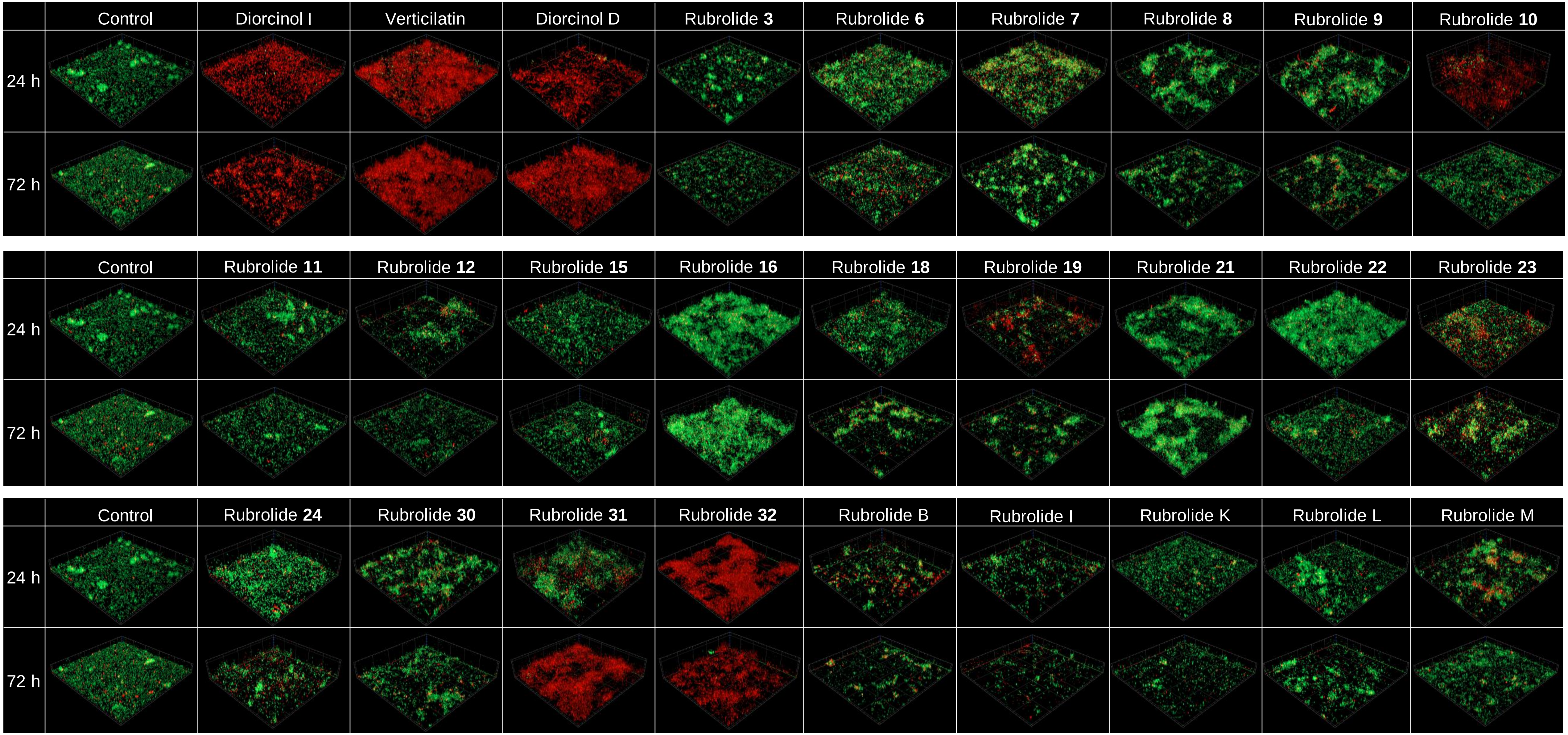

FIGURE S3: Synthetic diorcinols and rubrolides alter biofilm structure of *S. maltophilia* K279a. The biofilm architecture of *S. maltophilia* K279a grown in presence of 100 mg L^{-1} of the diorcinols or rubrolides were analyzed at different time points via CLSM after a live/dead-staining. Cells were grown in µ-slides at 28 °C in 10 % LB medium. Red: dead cells. Green: living cells. Images represent an area of 100 µm x 100 µm of the biofilm. Control: Biofilm grew in presence of 1 % DMSO.

FIGURE S3

FIGURE S4

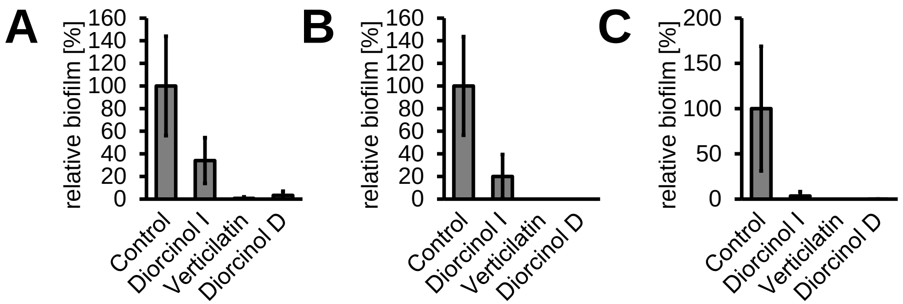

FIGURE S4: Reduced cell viability of biofilms grown in presence of diorcinols. CFU/ml of *S. maltophilia* K279a (**A**), SKK55 (**B**) and 454 (**C**) biofilm cells grown for 24 hours in 10 % LB at 28 °C in presence of 100 mg L^{-1} diorcinol I, verticilatin and diorcinol D was determined. Cells grown with 1 % DMSO were used as control. Error bars indicate standard deviation.

SUPPLEMENTARY FIGURES LEGENDS

FIGURE S1: Structures of all diorcinols and rubrolides used in this study. Structures are grouped into antibiofilm effective (Green; $I_{a\text{-}e}$) and non-effective (Purple; $II_{a\text{-}g}$) according to hierarchical clustering (Figure 2A).

FIGURE S2: Diorcinols and rubrolides reduce the biofilm formation of *S. maltophilia* K279a. The planktonic growth (light grey) and biofilm formation (dark grey) of *S. maltophilia* K279a grown in presence of 100 mg L^{-1} of the diorcinols and rubrolides were analyzed in relation to the control via crystal violet staining. Planktonic cells were grown at 28 °C in 10 % LB medium. C = Control (K279a grown with 2 % DMSO). Error bars indicate standard deviation. Data represent mean values of at least 3 replicates.

FIGURE S3: Synthetic diorcinols and rubrolides alter biofilm architecture of *S. maltophilia* K279a. The biofilm architecture of *S. maltophilia* K279a grown in presence of 100 mg L^{-1} of the diorcinols or rubrolides were analyzed at different time points via CLSM after a live/dead-staining. Cells were grown in µ-slides at 28 °C in 10 % LB medium. Red = Dead cells. Green = Living cells. Images represent an area of 100 µm x 100 µm of the biofilm. Control: Biofilm grew in presence of 1 % DMSO.

FIGURE S4: Reduced cell viability of biofilms grown in presence of diorcinols. CFU/ml of *S. maltophilia* K279a (**A**), SKK55 (**B**) and 454 (**C**) biofilm cells grown for 24 hours in 10 % LB at 28 °C in presence of 100 mg L^{-1} diorcinol I, verticilatin and diorcinol D was determined. Cells grown with 1 % DMSO were used as control. Error bars indicate standard deviation.

SUPPLEMENTARY TABLES LEGENDS

TABLE S1: *S. maltophilia* clinical and environmental isolates used in this study.

TABLE S2: Regulated genes of *S. maltophilia* K279a and 454 after treatment with diorcinols.

TABLE S3: Regulated genes of *S. maltophilia* K279a and 454 after treatment with rubrolides.

TABLE S4: Shared up and downregulated genes in *S. maltophilia* K279a and 454 treated with diorcinols and rubrolides.
